# Whole genome identification of *Mycobacterium tuberculosis *vaccine candidates by comprehensive data mining and bioinformatic analyses

**DOI:** 10.1186/1755-8794-1-18

**Published:** 2008-05-28

**Authors:** Anat Zvi, Naomi Ariel, John Fulkerson, Jerald C Sadoff, Avigdor Shafferman

**Affiliations:** 1 Israel Institute for Biological Research, Ness Ziona 74100, Israel; 2 Aeras Global TB Vaccine Foundation, Rockville, MD, USA

## Abstract

**Background:**

*Mycobacterium tuberculosis*, the causative agent of tuberculosis (TB), infects ~8 million annually culminating in ~2 million deaths. Moreover, about one third of the population is latently infected, 10% of which develop disease during lifetime. Current approved prophylactic TB vaccines (BCG and derivatives thereof) are of variable efficiency in adult protection against pulmonary TB (0%–80%), and directed essentially against early phase infection.

**Methods:**

A genome-scale dataset was constructed by analyzing published data of: (1) global gene expression studies under conditions which simulate intra-macrophage stress, dormancy, persistence and/or reactivation; (2) cellular and humoral immunity, and vaccine potential. This information was compiled along with revised annotation/bioinformatic characterization of selected gene products and *in silico *mapping of T-cell epitopes. Protocols for scoring, ranking and prioritization of the antigens were developed and applied.

**Results:**

Cross-matching of literature and *in silico*-derived data, in conjunction with the prioritization scheme and biological rationale, allowed for selection of 189 putative vaccine candidates from the entire genome. Within the 189 set, the relative distribution of antigens in 3 functional categories differs significantly from their distribution in the whole genome, with reduction in the Conserved hypothetical category (due to improved annotation) and enrichment in Lipid and in Virulence categories. Other prominent representatives in the 189 set are the PE/PPE proteins; iron sequestration, nitroreductases and proteases, all within the Intermediary metabolism and respiration category; ESX secretion systems, resuscitation promoting factors and lipoproteins, all within the Cell wall category. Application of a ranking scheme based on qualitative and quantitative scores, resulted in a list of 45 best-scoring antigens, of which: 74% belong to the dormancy/reactivation/resuscitation classes; 30% belong to the Cell wall category; 13% are classical vaccine candidates; 9% are categorized Conserved hypotheticals, all potentially very potent T-cell antigens.

**Conclusion:**

The comprehensive literature and *in silico*-based analyses allowed for the selection of a repertoire of 189 vaccine candidates, out of the whole-genome 3989 ORF products. This repertoire, which was ranked to generate a list of 45 top-hits antigens, is a platform for selection of genes covering all stages of *M. tuberculosis *infection, to be incorporated in rBCG or subunit-based vaccines.

## Background

Mycobacterium tuberculosis (Mtb), the causative agent of tuberculosis (TB), remains a major health threat. Each year, 8 million new TB cases occur and 2 million individuals die of TB [1]. Moreover, it is estimated that one third of the population is latently infected with Mtb, of which ~10% will develop active disease during lifetime. The development of active TB occurs when the balance between natural immunity and the pathogen changes (e.g. upon waning of protective immune response during adolescence and in HIV patients, [2]). In addition, at present ~50 million individuals are probably infected with multi drug-resistance (MDR) strains of Mtb (WHO, 2006), rendering antibiotic treatment difficult.

The current vaccine, introduced over 80 years ago, is the live attenuated bacterium *Mycobacterium bovis *Bacillus Calmette-Geurin (BCG), designed as a prophylactic vaccine for pre-infection administration. BCG is known to protect young children against severe forms of TB however it does not efficiently and consistently protect adults against the most prevalent form of the disease, namely, pulmonary TB (variable protective efficacy ranges from 0% to 80%), nor does BCG offer protection from establishment of latent TB and subsequent reactivation [3-9].

In principle, current putative vaccination strategies against TB can be divided into two groups: (a) prophylactic vaccines (aimed at disease prevention), based on BCG with or without antigens secreted by replicating bacteria recognized during the early stage of infection (e.g. ESAT-6 or Ag85A/B), or protective mutants (live attenuated BCG substitutes) and (b) as yet undefined post-exposure vaccines (boosting BCG) aimed at elimination/containment of latent TB and prevention of reactivation. Ideally, a prime-boost approach comprising of a prophylactic vaccine with subunit, viral vectored or DNA-based vaccines, based on late-stage antigens induced in the dormant stage (transition from replicating to non-replicating stage, latency antigens) or resuscitation/reactivation stage, should have maximum impact on all stages of Mtb infection [2,6,10-15].

In the past few years there have been important breakthroughs in the development of improved prophylactic TB vaccines. Novel vaccine candidates, mostly selected as single gene products, include: rBCG vaccines (e.g. rBCG30 – expressing Ag85B or ΔureCHly+rBCG – an urease deficient strain expressing listeriolysin), virus-based recombinant vaccines (e.g. MVA85A – Rv3804c expressed in replication-deficient vaccinia virus), live attenuated Mtb strains and subunit vaccines comprising of dominant secreted antigens which could boost the immune response after priming with BCG (e.g. Mtb72F, Ag85B-ESAT-6 fusion), (reviewed by [1,4-7,10,16]).

Post-exposure vaccine development requires identification of gene products participating in adaptation of Mtb to the intracellular habitat as Mtb changes from replication to dormancy or from dormancy to resuscitation. Moreover, in the absence of effective prediction models and animal models assessing protective immunity, evaluation of a large number of individual antigens remains laborious [12]. *In vitro *gene expression studies under conditions which mimic dormancy and/or reactivation constitute the major source of information. Availability of whole genome sequences of diverse mycobacterial strains in conjunction with data from global analyses such as whole-genome DNA microarray and proteomic technologies have been the subject of intensive research in the recent years, both at site of pulmonary TB and in *ex vivo *macrophages [11,17]. To identify genes expressed specifically during latency, different *in vitro *conditions have been suggested to model the harsh environment within macrophages or granulomas. These include oxygen deprivation, nutrient starvation and iron limitation. Together with expression studies in lungs/granulomas, in phagocytized bacteria inside activated macrophages or in murine/guinea pig infection models, these datasets provide better understanding of the transition of bacteria through stages of active multiplication, dormancy and resuscitation. Based on bioinformatic studies and analysis of multiple dormancy-related datasets, novel drug targets against the dormant phase of Mtb infection have been recently identified [18].

Several attempts have been made to modify the immunogenicity or antigenicity of BCG by generating recombinant strains expressing cytokines, pore-forming listeriolysin/perfringolysin, immunodominant antigens or additional antigens missing from the genome of the avirulent *M. bovis *BCG genome. Over 200 genes located in 14 gene segments assigned to defined regions of difference (RDs) are missing in the vaccine strain. In principle, potency of BCG vaccines could be improved by supplementing with missing immunodominant RD genes; however critical evaluation is required since RD regions are associated with virulence. Indeed, when BCG was supplemented by the classical RD-1 antigen ESAT-6 (with or without Ag85B [19]), enhanced protection was observed in mice however the recombinant strain was more virulent as compared to the wild-type. In a more recent study, Kalra & Grover [20] demonstrated the enhanced prophylactic potential of selected combinations of RD antigens supplementing BCG, improving protection in aerosol exposed mice.

Mtb is an intracellular pathogen and as such cell mediated immunity rather than antibody-mediated immunity is essential for the control of bacterial replication and subsequent protection against TB [7]. It is thought that a coordinated response of the cellular immune response is fundamental to the protective immunity [21-23], including both CD4 and CD8 T-cells and several cytokines such as IFNγ and TNFα. It has been suggested that CD4 T-cells are mainly crucial during the early phases of infection, while the CD8 T-cells play an important role in the chronic phase of the disease [21-23], however little is known about antigen-specific human T-cell responses against persisting mycobacteria, particularly in the context of latent infection and protection against disease, with the exception of some products of the DosR regulon [24,25]. Therefore, rational selection of sequences that may function as T-cell epitopes in vaccine formulations is crucial. Relatively few human CD8 T-cell epitopes have been found by conventional methods. The availability of the genomic sequence [26] provides new horizons in analyzing the potential immunome of the bacilli, using *in silico *identification of CTL binders. Several prediction screens of Mtb T-cell epitopes were reported to date [25,27-32], nevertheless these analyses were mostly restricted either to few MHC alleles or to a limited number of preselected subset of genes.

In this study, we have combined multiple published datasets from: (a) gene expression and DNA microarray experiments mimicking conditions leading to dormancy or at the dormant state; (b) genome-wide insertional mutagenesis examining gene essentiality under different conditions; (c) genes expressed in macrophages under conditions mimicking persistence; (d) genes expressed in lungs/granulomas; (e) expression under iron limiting conditions; (f) identification of genes products reported to elicit humoral and cellular response or potential vaccine candidates; (g) *in silico *identification of T-cell epitopes by dedicated epitope-mapping algorithms and databases compiling relevant experimental data. We designed a whole-genome scoring, ranking and prioritization algorithm and used it to analyze the combined dataset. Accordingly, we selected a set of 189 putative vaccine candidate proteins covering all disease phases, from which we further derived a shorter list of 45 top-ranking antigens for vaccine studies.

## Methods

Genome sequences and annotations of *Mycobacterium tuberculosis *H37Rv [GenBank: AL123456] were downloaded from the NCBI [33]. Sequence similarity searches were conducted using the Blast algorithm, vs. the non-redundant (nr, NCBI) database. Comparisons against secondary databases of domains and motifs were performed as follows: CDD [34], SMART [35], Pfam [36], Interpro [37], Prosite [38]. Secretion signals were analyzed by SignalP [39,40] for the secretion via the SecA pathway, and TATfind for searching of the Twin-Arginine Translocation (Tat) motif of the TAT pathway [41]. Transmembrane helical segments were predicted by Tmpred [42]. The database of bacterial lipoproteins, Dolop [43] was searched for putative lipoproteins. Predictions of CTL epitopes were carried out using the NetCTL integrative approach [44], which combines predictions of MHC class I binding, TAP transport efficiency, and proteasomal cleavage. The analysis was conducted for each of 12 HLA supertypes. A threshold of 1.25 was set on the combined prediction score. The results were parsed by in-house perl scripts. Immune epitope information was complemented by querying the IEDB database [45] for documented B- and T-cell epitopes. The following Mtb-related servers and databases were screened for additive relevant data: TB-sgc – The TB Structural Genomics Consortium [46], Tuberculist-the database on *Mycobacterium tuberculosis *genetics [47], TBDB – an integrated platform for TB drug discovery [48], MTBreg – The Database of Conditionally Regulated Proteins in *Mycobacterium tuberculosis *[49] and BioHealthBase – the Biodefense and Public Health Database [50]. Functional categories provided for the selected ORF products were as implemented in [26,51], and their assignment to each of the antigen was derived from the Tuberculist database (see above). Classes/phases of Mtb infection were assigned as well to the selected antigens: the well established classical antigens, DosR-regulated antigens (as reported in [52,53]), reactivation antigens (antigens listed by Talaat and his coworkers [54], as well as documented putative implications of individual antigens), and the 5 known resuscitation antigens [55]; antigens not belonging to any of the above classes, were denoted as "Others". p-values were calculated using the binomical distribution of frequencies.

## Results and Discussion

**Figure 1:**
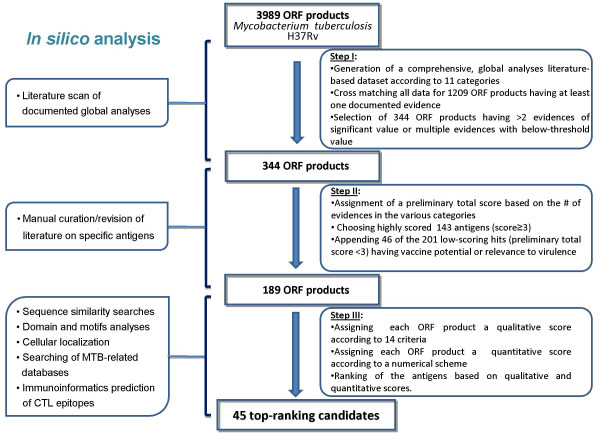
**Flowchart of the selection procedure – data mining and bioinformatic analyses**. A schematic presentation of the reductive approach applied for the whole-genome selection of Mtb vaccine candidates.

The strategy for whole genome-based selection of candidate genes to be included in a vaccine platform, consisted of: (1) compilation of documented data originating from global analyses pertaining to criteria relevant to vaccine and to *M. tuberculosis *(Mtb) pathogenesis (2) selection of a subset of genes for further evaluation, based on the accumulated data, by cross matching the data in the different criteria; (3) bioinformatic analyses aimed at both further characterization of the candidate genes, in terms of annotation, gene context and cellular localization and; immunoinformatic analysis conducted for the prediction of T-cell epitopes; (4) development and application of a ranking scheme, based on qualitative and quantitative measures, as a tool for prioritizing the selected candidates. A schematic presentation of the various steps, as well as the reductive scheme generating a list of best-hit antigens, is given in Figure 1.

### Generation of a comprehensive, literature-based dataset

The first step towards the selection of vaccine candidates consisted of accumulating published experimental data, by an extensive literature scan of documented analyses, with emphasize on global analyses. Target genes to be involved in a future vaccine are either ORF products which have the potential to elicit an immune response (humoral and/or cellular), and/or involved in various manifestations and phases of the infection and/or virulence (as a basis for an attenuated strain). Accordingly, we assembled all evidences into the following general 11 categories (Table 1):

**Table 1:** Information sources for the knowledge dataset used in this study.

(I) Literature-derived evidences:			
**Category**		**Reference**	**Experimental model/method**

**Macrophages**	***Growth/Survival***	Sassetti *et al*., 2003	Genes essential for growth (in strains H37Rv & BCG) (TraSH, microarray)
		Rengarajan *et al*., 2005	Genes necessary for survival in macrophages (TraSH, microarray)
		Stewart *et al.*, 2005	Screening of mutants unable to inhibit phagosome acidification (STM, microarray analysis)
		Rosas-Magallanes *et al*., 2007	Screening of mutants attenuated in human macrophages (STM)
	***Expression profile***	Monahan *et al*., 2001	*M. bovis *BCG protein expression in macrophages (human cell line), as compared to growth in culture media or conditions of heat shock (1,2-DE, proteomic analysis)
		Fisher *et al*., 2002	Genes induced following *in vitro *acid shock (microarray, RT-PCR)
		Schnappinger *et al*., 2003	Differential transcriptome of genes in the phagosome, in comparison to their expression in culture (microarray, RT-PCR)
		Talaat *et al.*, 2004	Comparison of H37Rv expression profile between growth in lungs of BALB/c vs. macrophages (microarray, RT-PCR)
		Cappelli *et al*., 2006	Comparison of H37Rv gene expression in human macrophages vs. synthetic medium (microarray, RT-PCR)
**MprAB**	***Regulation***	He *et al*., 2006	Genes upregulated by MprA (in SDS treated culture); (microarray RT-PCR)
**Hypoxia**	***Expression profile***	Voskuil *et al*., 2003	Expression profile at low O2 and low concentrations of NO (inhibitor of aerobic respiration); dormancy regulated gene set; mostly overlaps with Sherman; microarray
		Sherman *et al*., 2001	H37Rv gene expression under hypoxia (from ambient to 0.2% O_2 _for 2 h)
		Schnappinger et al., 2003	Analysis of mutants deficient in NO synthase
**Reactivation**	***Expression profile***	Talaat *et al*., 2007	Difference in expression in BALB/c vs. broth after incubation with dexamethasone (microarray)
		Tufariello *et al*., 2006	Effect of rpf deletions on persistence and reactivation in mouse
**Dormancy**	***Expression profile***	Voskuil *et al. *2003	Expression profile at low O2 and low concentrations of NO; dormancy regulated gene set; mostly overlaps with Sherman; microarray
		Voskuil *et al*., 2004	Transcription profile in non-proliferating conditions; genes induced under oxygen-depleted conditions (nrp-non-replicating persistence)
		Starck *et al*., 2004	Proteome comparison of aerobic and anerobic conditions (MTB Harlingen strain)
**Lungs**	***Expression profile***	Fenhalls *et al*., 2002	Expression of genes in human tuberculous granulomas (in situ hybridization)
		Shi *et al*., 2003	Transcription pattern of 6 H37Rv genes in mouse lungs (RT-PCR)
		Shi *et al*., 2004	Transcription pattern of H37Rv major secreted antigens in mouse lungs (RT-PCR)
		Dubnau *et al*., 2005	Expression of genes during infection in mouse lungs vs. medium (promoter trap)
		Rachman *et al*., 2006	Identification of genes expressed during pulmonary TB; transcription profile from clinical lung samples (granuloma vs. *in vitro*) (microarray)
		Tufariello *et al*., 2006	rpf gene expression in the lungs of infected mice
	***Growth/Survival***	Lamichhane *et al*., 2005	Genes required for in vivo survival im mouse lungs (microarray screening of transposon mutants)
		Jain *et al*., 2007	Mutants tested for lung implantation in survival in guinea pigs and mouse aerosol models
**Acr**	***Co-regulation***	Florczyk *et al*. 2003	Identification of a 18-bp palindromic sequence motif
**Secreted**		Gomez *et al*., 2000	*in silico *identification of proteins harboring signal peptides but lacking membrane anchoring moieties.
**Immunogenicity**	***B-cell response***	Brusasca *et al*., 2001	Antibody response to 6 H37Rv RD1 proteins in guinea pigs and sera from pulmonary TB patients
		Yeremeev *et al*., 2003	Elicitation of B-cell response in mice immunized with rpf proteins (H37Rv)
		Weldingh *et al*., 2005	Seropotential of 35 proteins, tested by response with sera of TB patients
		Amor *et al*., 2005	Seroreactivity of MTB specific proteins previously predicted as secreted
	***T-cell response***	Cockle et al., 2002	Immune response in cattle against 13 ORFs (RD1, RD2 and RD14 antigens).
		Vekemans *et al.*, 2004	Profile of immune response in healthy and TB patients against a series of mycobacterial antigens
		Mustafa *et al*., 2006	Characterization of Th1 cell reactivity with RD1 antigens and peptides
**Iron-regulated**	***Regulation***	Rodriguez *et al*., 2002	Identification of genes induced by iron and by the iron-dependent regulator IdeR – comparison of H37Rv and ideR-mutant strains (microarray)
**Vaccine**	***Immunization/protection***	Mollenkopf *et al*., 2004	DNA vaccine candidates preselected by comparative proteomics (present in MTB, absent from BCG) evaluated for their protective potential (aerosol challenge of H37Rv, mice)
		Vipond *et al*., 2006	DNA vaccine candidates chosen by supporting data, such as virulence-associated, level of expression, growth in various conditions etc. (aerosol challenge of H37Rv, guinea pigs)
		Roupie *et al*., 2007	DNA vaccine candidates chosen from the DosR regulon (on the basis of strong T-cell responses in infected humans), evaluated for their immunogenicity potential (mice immunizations).

(II) *in silico*-based evidences:			

**Category**		**Source of information/analyses**	

**Cell wall**	***Membranal and anchored***	Assignment of ORF products as membrane-attached, by:	
		(1) Prediction of membrane-spanning regions by TMpred	
		(2) Inference from annotation and/or domain analysis	

**Repeats**		Inference from annotation and/or domain analysis	

**T-cell immunogenicity**	***MHC class I and class II binders***	Compilation of experimental and predicted data from:	
		(1) Screening of the public repository database of immune epitope data (IEDB)	
		(2) Particular experimental evidences from the literature	
		(3) Literature-derived predicted T-cell epitopes	
		(4) Prediction of CTL epitopes by an integrative approach (NetCTL)	

#### Macrophages

Numerous comprehensive studies related to the infection of the macrophages by the bacilli were conducted, aiming at identifying the genes involved in processes as adhesion to the host cell, phagocytosis-mediated entry and survival in the macrophage, adaptation to the environment and resistance to both phagocytosis and lysis, and to free radicals. Different approaches were applied to characterize both the profile of expression at various relevant conditions and the growth/survival of wild-type Mtb and selected mutants. These include subtractive hybridization, transcriptomics, proteomics and mutagenesis. In our screen, we included representative studies covering the abovementioned aspects, as detailed in Table 1[56-64].

#### MprAB

This two component system is one of two major families of transcriptional regulators necessary for stress adaption processes. The system is induced in response to various stress conditions and was shown to be required for growth *in vivo *during the persistent stage of infection. Comparative DNA microarray analysis of Mtb H37Rv and an *mprA *mutant strain enabled to profile the gene expression in each of the strains and to identify 221 genes which were presumably regulated by MprA under stress conditions [65].

#### Hypoxia

One of the well established models of the tuberculi non-replicating dormant state, is the Wayne model [66], which is based on culturing *Mycobacteria *in decreasing concentrations of oxygen, mimicking the environment in which the bacteria transform to a non-replicating form, residing in granulomas, which are of hypoxic nature. In this respect, growth under low O_2 _and NO concentration or under depletion of NO synthase are the major conditions implemented to identify the expression profile related to hypoxic conditions. Sherman and his coworkers [67] used a whole genome microarray to identify 101 genes whose expression is significantly altered by hypoxic conditions, of which 47 are induced. A similar approach was followed by Voskuil and coworkers [52], resulting in a set of 48 genes affected by low O_2 _concentration or by the presence of nontoxic concentration of NO (known to inhibit bacterial respiration). Likewise, mutants deficient in NO synthase were used to profile the genes expressed during hypoxia-like conditions [62].

#### Reactivation

Very little is known about the genetic basis and the signals which are involved in the reactivation from the dormant state of the mycobacterium. In an attempt to decipher the factors which govern the phase of reactivation, some researchers adopted an immunossupressive model, in which reactivation of latent infection occurs following treatment with the immunossupressive reagent dexamethazone [68,69]. Using this model, Talaat and coworkers [54] recently identified a total of 174 genes that were up-regulated during the reactivation phase of tuberculosis. A family of 5 proteins, denoted resuscitation promoting factors (Rpfs), has been shown to be involved in regulating mycobacterial growth [55,70]. Single deletions of the Rpf members were used to determine the effect of these proteins on the kinetics of reactivation [71].

#### Dormancy

The shifting to the non-replicating persistence state ("dormancy") of the bacteria is accompanied by modulation of expression of genes related to the adaptation to the non-replicating conditions. The adaptive processes include: starvation of essential nutrients, cessation of growth in stationary phase, and depletion of oxygen. Of these, the later is the most investigated and constitutes the basis for the *in vitro *model used in dormancy studies. The set of genes found to be induced by hypoxia, nitric oxide and adaptation to the non-replicating conditions, under the regulation of DosR (Dormancy survival regulator, Rv3133c), were denoted as the dormancy/DosR regulon [52,53]. Transcription profiles were determined by a whole-genome comparative DNA microarray analysis of the exponential growth at time points in the stationary phase, as well as under specific non-replicating persistent conditions of low oxygen and nitric oxide [52,53,62]. In a separate analysis, the differential expression of genes under aerobic and anaerobic conditions was deciphered by a proteome (2D-PAGE) analysis, revealing proteins which were unique or more abundant in the anaerobic conditions [72].

#### Lungs

As the first point of entry of the *M. tuberculosis *upon infection, following implantation the bacteria reside and replicate in granulomas in the lungs. Various strategies were employed to depict the pattern of expression both in human granulomas and in guinea pig and mouse lungs, and these revealed an extensive list of genes necessary for growth and survival, as well as identification of genes expressed during infection in the lungs (detailed in Table 1, [17,71,73-78]).

#### Acr-coregulated

The 16 kDa α-crystallin (Acr) protein was shown to be induced and required inside macrophages and highly expressed in the presence of NO, low O_2 _concentrations and in the stationary phase ([79-81] and references therein). The Acg gene product was later identified as a novel macrophage-induced gene, whose expression is co-regulated with that of *acr *[79]. Aiming at identifying other genes which are under the same regulation, an *acr-*coregulated gene (ACG) family was denoted, following the identification of a conserved sequence motif found in the promoter region of the 15 family members [82].

#### Secreted

Proteins which are exported from the cytoplasm and either secreted to the milieu or anchored to the cell wall, are of a major importance as targets of the immune system, in view of their exposure in the host. The secretion pathways in *M. tuberculosis *are less established than in other bacilli, in particular those implicated in virulence factor secretion. In addition to the classical pathways (the SecA and TAT systems), the pathogen harbors a unique virulence-related secretion system – ESX system and probably additional systems which remain to be identified. Identification of secreted proteins by a whole genome global analysis was conducted by Gomez and coworkers [83]. A predictive approach of all proteins harboring a secretion signal of the classical SecA pathway, but lacking membrane spanning segments, was carried out, resulting in the identification of 52 proteins, the location of which was further confirmed by fusion to a marker of subcellular localization.

#### Immunogenicity

Data on immune response was compiled from global analyses covering both elicitation of humoral and cellular responses following vaccination of model animals, as well as seroreactivity profiles of selected antigens with sera from healthy and TB patients (see Table 1 and data pertaining to individual ORF products, in the text, [30,84-89]).

#### Iron regulated

As for many other pathogens, *Mycobacterium tuberculosis *infection is dependent on the availability of iron in the depleted macrophage, and therefore iron sequestration from the host is necessary for virulence [90,91]. Iron is also known to influence both the innate and adaptive immune responses to Mtb [92]. Failure to assemble the iron acquisition machinery or to repress iron uptake has deleterious effects for Mtb [90,91]. In addition to expressing iron-uptake systems during iron deficiency, Mtb displays several changes in gene expression in response to iron availability. All changes taking place during the response to iron deficiency are controlled by iron-dependent regulatory networks. The IdeR gene product is a dual functional regulator controlling transcription of genes involved in iron acquisition, iron storage and macrophage survival [93,94]. *ideR *is an essential gene in *M. tuberculosis *and its deletion results in deregulated siderophore biosynthesis and sensitivity to oxidative stress [94]. The transcription profile of genes whose expression is modulated by iron levels was mapped by a DNA microarray analysis, and revealed 155 genes which were iron-regulated; one third of these were regulated by IdeR [94].

#### Vaccine

Data pertaining to vaccine potential of the antigens was derived from publications describing global analyses of preselected antigens for their potential to induce an immunoprotective response [25,95-97]. These include mostly evaluation of DNA vaccine candidates, as listed in Table 1. For this specific category, a large number of evidence was also derived from information pertaining to particular antigens studied both as DNA or protein vaccine candidates [14,20,24,25,71,84,98-141], among which are obviously the well established classical antigens.

### Selection of a dataset of ~200 vaccine candidates

Of the total 3989 ORF products, 1209 had documented evidence in at least one of the sources listed in Table 1. Cross-matching of the compiled data for all 1209 ORF products, allowed for a preliminary selection of 344 antigens, for which either more than two values above the threshold exist, or, alternatively, one value above the threshold together with at least two other values with lower values (below the threshold) were present. The threshold for each literature source was set as the median of the experimental values extracted from the particular literature source.

The literature sources which were used to generate the dataset (Table 1) were grouped within each category (e.g. all 9 studies in the Macrophage category were treated as a single piece of evidence). Following the threshold selection described above (step I in Figure 1), each ORF product was assigned a "+" sign in a given category if experimental data has been reported at least in one study. Summation of the "+" signs across all categories, for each ORF product, produced a total, un-weighted score, with a maximal possible total score of 11 (according to the number of categories). Among the 344 antigens, 143 had a relatively high total score of 3–8. Of the remaining 201 low scoring antigens, 46 had documented information related to vaccine potential and/or a critical role in the pathogenesis traits of the bacilli. As such, these 46 antigens were added to the 143 high scoring antigens (having a total score ≥ 3), generating the final list of 189 candidates for further evaluation (Additional file 1: List of 189 selected antigens).

### Bioinformatics analysis of the 189 vaccine candidates

Inspection of the list of 189 candidates reveals that, according to the annotation deposited at the NCBI, 69 antigens have no assigned function. In an attempt to further characterize the candidate genes, we followed an in-depth bioinformatic analysis which was aimed at revising the existing annotation, as well as determining the cellular localization and the presence of secretion signals, and protein repeats patterns. The revised annotation was thus based on sequence similarity searches against updated databases, and domain/motif assignment following querying secondary databases (see: Methods). Also, Mtb-related servers were screened to extract function-related information (see: Methods). Additive information was derived from the accumulating data in the literature, and specifically on functional characterization of particular genes or their mycobacterial orthologs. These *in silico *analyses and data mining resulted in the assignment of a putative function to 60% of the 69 candidates with unknown function, and the addition of further functional details to 15 candidates (Additional file 1: List of 189 selected antigens).

### Immunoinformatic analysis of the 189 vaccine candidates

As mentioned above, the significance of the cellular immune response in protection against intracellular pathogens in general and Mtb in particular, is well documented. We therefore conducted a comprehensive mapping of the T-cell immunity potential of the selected antigens, by compiling both the results of an *in-silico *search for putative T-cell epitopes and experimental published data.

Using NetCTL as an integrative approach for prediction of 9-mer CTL epitopes [44], we analyzed the 189 proteins for the presence of MHC binding peptides. The analysis was conducted on 12 HLA supertypes, representing ~120 human MHC alleles and providing a population coverage of 99.8% worldwide. For our large scale analysis, we have chosen a relatively high combined score threshold of 1.25, which gives preferentiality to true binders. At this threshold, the specificity is 0.993 and the sensitivity is 0.54 (as reported by the NetCTL server). Most potent candidates were considered as those having binders for as many supertypes (this argument was further used to rank the antigens at a later stage, see below). Compared to *in silico *studies of the Mtb antigens immunomic potential [25,27-29,31,32,125], the analysis described herein is inclusive both with respect to its scope and to the number of HLA alleles covered.

In addition to the theoretical analysis, experimental evidences for T-cell epitopes were retrieved both from the IEDB database [45], by querying each of the antigens as well as data for cellular immunoreactivity of individual gene products from the extensive study of Leyten *et al. *[24] and other literature sources [24,25,84-86,88,99,102,106,115,119,124,127,142-144]. The compiled T-cell immunity data was added as yet an additional criterion for further evaluation and ranking of the 189 antigens, leading to a total of 14 criteria.

### Assignment of a qualitative score to the 189 vaccine candidates

The compilation of the experimental data with the bioinformatic and immunoinformatic analyses, together with biological reasoning resulted in a comprehensive knowledge-based dataset of the 189 vaccine candidates (Additional file 2: "Raw data of the 189 selected antigens"), according to a total of 14 criteria (Table 1). In order to rank the list, and further prioritize the antigens, we developed a scheme which is based on both a qualitative and a quantitative score calculated for each antigen. The scores are calculated according to 14 criteria, encompassing the 11 literature-based categories as well as T-cell immunome data, cell wall and repeats-derived information. The first scoring iteration assigns an equal weight to each criterion. A "+" sign is indicated for each indication in each of the criteria, and an arithmetic score is calculated, by summing the "+" signs (See Additional file 3: 'Qualitative scores for 189 selected antigens'). The scores obtained ranged between 9-1, exhibiting a normal distribution of number of antigens per scores, as follows: scores 9–8: 11.6%; scores 7–6: 32.8%; scores 5–4: 45.5% and; scores 3-1: 12%. This rather naïve scoring is of value in rendering a preliminary ranking, which allows to limit the bias that may arise in favor of antigens with an extensive coverage in the literature. Nevertheless, it suffers from some inherent limitations: (1) as described above, an equal weight was assigned to each criterion, yet their differential relevance to vaccine development is not reflected; (2) any data in a specific criterion contributed equally to the total qualitative score, regardless of the relative potency of the actual result reported in the publication from which the data was extracted, or for data obtained from our analyses. (3) in spite of a broad range of qualitative scores, their distributions reveal large clusters of genes. To note, groups of 17 and 19 genes harbor a qualitative score of 8 and 7, respectively, while a group of 44 genes have a qualitative score of 6. This implies that in order to prioritize the equally-scored antigens within the groups, we had to further refine our scoring system and apply additional measures.

### Employing a numerical scheme for the assignment of quantitative scores

To address the above-described limitations of the qualitative measure, we introduced weighted internal scores to each of the 14 criteria, as presented in Table 2. The actual data for determination of the internal scores was extracted from the relevant experimental studies. The internal score scaling was based on the number of sources and/or the intensity of the results which contributed to the particular criterion. Accordingly, the maximal internal score was 2 for most criteria, except for Macrophage and Vaccine, which were up-weighted due to their significance/relevance to vaccine development, by giving a maximal internal score of 3. These scoring rules were also useful for the final ranking among high scoring genes. The final quantitative score calculated for each ORF product, is the total of the internal scores assigned in each of the 14 criteria for a particular ORF product. As expected, the quantitative scores allowed dissecting the large groups of equally qualitatively scored antigens, and ranking further the candidates. These scores can be used as a tool for further trimming down the number of vaccine candidates, and selecting for the top-hit targets. This process generated a list of 55 antigens with a qualitative score value of 6 and above. Of this list, 10 antigens were predicted to harbor more than 5 membrane-spanning domains (Rv0286, Rv0290, Rv0450c, Rv0754, Rv1196, Rv1348, Rv1736c, Rv1737c, Rv1997, Rv2123). Although potentially valuable vaccine targets, these antigens were removed from the list due to the potential technical problems which may occur during cloning and production of such recombinant proteins. Consequently, a list of 45 top ranking antigens is provided (Table 3). In this list, the antigens are sorted by their qualitative score and consecutively – by their quantitative score. According to this type of ranking, the antigens are clustered into groups, by limiting both the qualitative and the quantitative lower value scores in a certain group. For instance, Group I of antigens includes candidates having a qualitative score of 8 and above, provided that the quantitative score is not lower than 12. According to such an approach for ranking, the antigens were delineated into three groups: the first includes the 12 top best-hit antigens both in terms of qualitative and quantitative scores and the following second and third groups comprise of 20 and 13 antigens, respectively (Table 3). There are other modes of sorting which could be considered as well, giving more weight to the quantitative score vs. the qualitative score and vice versa. It should be mentioned though, that using these different ranking approaches had marginal consequences on the grouping, affecting mainly those antigens at the edges of each of the groups.

**Table 2:** Numerical internal scores.

**Criterion**	**Internal score**	**Maximal score**
Macrophage	(0) no evidence	**3**
	(1) significant evidence from one source	
	(2) significant evidence from two sources	
	(3) significant evidence from > two sources + high value	
MprAB	(0) no evidence	**2**
	(1) <3.0 fold expression	
	(2) >3.0 fold expression	
Hypoxia	(0) no evidence	**2**
	(1) 1 evidence	
	(2) >1 evidence, high values	
Reactivation	(0) no evidence	**2**
	(1) evidence from one source	
	(2) evidence from two sources	
Dormancy	(0) no evidence	**2**
	(1) low values	
	(2) high values	
Lung	(0) no evidence	**2**
	(1) evidence from one source	
	(2) multiple evidences (or Rachman/Jain source)	
*acr*-coregulated	(0) no evidence	**2**
	(2) up regulated	
Secreted	(0) no evidence	**2**
	(1) secreted	
	(2) secreted+virulence-related function	
B-cell immunogen	(0) no evidence	**2**
	(1) evidence from one source	
	(2) multiple evidences	
Iron regulated	(0) no evidence	**2**
	(1) low values	
	(2) high values	
Cell wall	(0) not related	**2**
	(1) general association (without tm)	
	(2) virulence-related function	
Vaccine	(0) no evidence	**3**
	(1) DNA/protein immunization, immune respone but no protection	
	(2) part of a multivalent construct, protection	
	(3) DNA/protein vaccine protection	
Repeats	(0) no repeats	**2**
	(1) repeats only	
	(2) repeats + virulence-related function	
T-cell		
Experimental	(0) no evidence	**2**
	(1) evidence from one source	
	(2) multiple different evidences	
Predictions	(0) 0< #supertypes <6	**2**
	(1) 6 < #supertypes <10	
	(2) 10<#supertypes <12	

**Table 3:** Top-ranking 45 antigens (sorted by quantitative and qualitative scores).

**Group**	**Rv #**	**Gene ^(a)^**	**Length (aa)**	**Annotation ^(a)^**	**Qual Total**	**Quant Total**
***I***	**Rv1738**		94	hypothetical protein	9	14
	**Rv2450c**	*rpfE*	172	probable resuscitation-promoting factor rpfE [transglycosylase]	9	14
	**Rv2623**	*TB31.7*	297	hypothetical protein TB31.7 [universal stress protein]	9	14
	**Rv1009**	*rpfB*	362	possible resuscitation-promoting factor rpfB [transglycosylase, C5 adhesion domain]	9	13
	**Rv0867c**	*rpfA*	407	possible conserved trans-membrane protein [transglycosylase, rpfA]	9	12
	**Rv2031c**	acr (α-crystallin)	144	heat-shock protein HspX (alpha-crystallin homolog) 14 kDa antigen Hsp16.3	8	14
	**Rv1886c**	*fbpB *(Ag85B)	325	secreted antigen 85-B FBPB (85-B) (mycolyl-transferase 85B)	8	14
	**Rv0288**	*esxH *(TB10.4)	96	Low Mw protein antigen 7 esxH (10 kDa antigen) CFP-7, TB10.4)	8	13
	**Rv2032**	acg	331	conserved hypothetical protein Acg [nitroreductase]	8	13
	**Rv2626c**		143	hypothetical protein [CBS pair – binding/regulation, euk]	8	13
	**Rv3873**	*PPE68*	368	PPE family protein [PPE68, RD1 T/B immunogen]	8	13
	**Rv2005c**		295	hypothetical protein [USP-like]	8	12
	**Rv3127**		344	hypothetical protein [possible nitroreductase]	8	12

***II***	**Rv1733c**		210	probable conserved trans-membrane protein	8	11
	**Rv1996**		317	hypothetical protein [USP]	8	10
	**Rv2389c**	*rpfD*	154	probable resuscitation-promoting factor rpfD [transglycosylase]	8	10
	**Rv0685**	*Tuf*	396	elongation factor Tu [iron-regulated]	8	9
	**Rv2628**		120	hypothetical protein	8	9
	**Rv1980c**	*mpb64*	228	immunogenic protein MPT64	7	13
	**Rv3804c**	*fbpA *(Ag85A)	338	secreted antigen 85-A FBPA (85-A) (mycolyl-transferase 85A)	7	13
	**Rv0079**		273	hypothetical protein	7	11
	**Rv3130c**	[*tgs1*]	463	hypothetical protein [diacylglycerol acyltransferase]	7	11
	**Rv3131**	[*bfnB*]	332	hypothetical protein [possible nitroreductase NfnB]	7	11
	**Rv0824c**	*desA1*	389	probable acyl [-acyl-carrier-desaturase desA1]	7	10
	**Rv1908c**	*katG*	740	catalase-peroxidase-peroxinitritase-T katG	7	10
	**Rv1174c**	[*sak5*]	110	Low Mw T-cell antigen TB8.4 [secretion antigen SA5K]	7	9
	**Rv1349**	[*irtB*]	579	probable drugs transport ATP-binding protein ABC transporter [ATM1 ABC siderophore-iron transporter]	7	9
	**Rv1813c**		143	hypothetical protein	7	9
	**Rv2006**	*otsB1*	1327	probable trehalose-6-phosphate phosphatase OTSB1	7	9
	**Rv2029c**	*pfkB*	339	possible phosphofructokinase (pfkB)	7	9
	**Rv2627c**		413	hypothetical protein	7	9
	**Rv2780**	*ald*	371	secreted L-alanine dehydrogenase ald (40 kDa antigen, TB43)	7	9

***III***	**Rv1884c**	*rpfC*	176	probable resuscitation-promoting factor rpfC [transglycosylase]	7	8
	**Rv2620c**		141	probable conserved transmembrane protein	7	8
	**Rv2744c**	*35kd-Ag* [*pspA*]	270	conserved 35 kDa Alanine-rich protein [phage-shock protein IM30]	7	8
	**Rv3875**	*esxA*	95	6 kDA early secretory antigenic target ESXA (ESAT-6)	6	11
	**Rv1926c**	*mpt63*	159	immunogenic protein MPT63 (16 kDa immunoprotective extracellular protein)	6	10
	**Rv2030c**		681	hypothetical protein [putative esterase/transferase]	6	10
	**Rv3132c**	*devs*	578	two component sensor histidine kinase DEVS	6	10
	**Rv3347c**	*PPE55*	3157	PPE family protein (PE55) [8 copies pentapeptide repeats]	6	10
	**Rv0467**	*icl*	428	isocitrate lyase(icl) [AceA]	6	9
	**Rv1130**	[*prpD*]	526	hypothetical protein [2 methyl-citrate dehydratase]	6	9
	**Rv1169c**	*PE11*	100	PE family protein (PE11) [triacyl glycerole lipase]	6	9
	**Rv1793**	*esxN*	94	putative ESAT-6-like protein ESXN (ESAT-6-like protein 5)	6	9
	**Rv2629**		374	hypothetical protein [peptide release factor erF1]	6	9

The antigens are sorted by the qualitative score (Qual Total) and subsequently by the quantitative score (Quant Total) (see text). Group I includes all antigens with a qualitative score 8 and above, provided that the quantitative score is not lower than 12. The rest of the antigens having a qualitative score of 8 and those having a qualitative score of 7 and a quantitative score not lower than 9 were clustered into Group II. Group III included antigens with qualitative scores of 7 (and quantitative score of 8) and 6 (with a quantitative scores 9 and up). (a) The Gene name and annotation are based on the data deposited at the NCBI, [GenBank: AL123456]. In square brackets: updated gene name and/or annotation, resulting from the analyses conducted in this study.

### Functional categories of the selected genes

**Figure 2:**
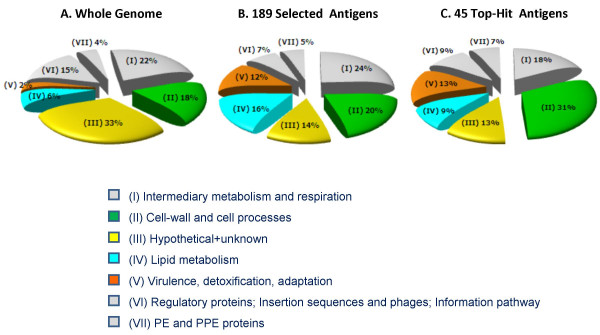
**Distribution of Mtb genes according to functional categories**. The categories are adapted from Cole and his coworkers [26]. For practical reasons, the categories of "Conserved hypothetical proteins" and "Proteins of unknown function" were joined (category (III) in the Figure). Category (VI) encompasses the functional classes which were either less abundant or non existent in the list of 189 antigens selected herein. The percentage of genes in each of the functional categories is provided, as retrieved from Camus and his coworkers for the whole genome (A) [51], and calculated for this study 189 selected antigens (B) and 45 top-ranking antigens (C).

According to the revised gene annotation and the accumulating data from the literature, the 189 selected antigens were assigned a functional category (listed in Additional file 1: List of 189 selected antigens) following the classification implemented previously for the Mtb complete genome sequence by Cole and coworkers (and re-annotated by Camus and co-workers, [26,51]). Figure 2 presents a graphic view of the gene distribution according to the functional categories, in the repertoire of the 189 selected genes. When compared to their fraction in the whole genome [51], it appears that the Conserved hypothetical proteins category is under-represented in the list of 189 selected antigens, while the Lipid metabolism and Virulence, detoxification and adaptation categories are over-represented in the list of 189 selected antigens. To note, this higher fraction is a *bona fide *observation, and not a result of antigens which were re-assigned to a new functional category following the updated annotation.

#### (1) Conserved hypothetical proteins

In the complete genome, 33% of the genes were assigned a functional category of either Conserved hypothetical proteins or Proteins of unknown function [51]. The proportion of this class of antigens is reduced to 14% in the list of 189 candidates presented in this study, resulting in 27 proteins for which the function remains unknown. This observation is evidently the consequence of the revision of annotation carried out in this study for the 189 selected genes, a process which, as detailed above, was based on cross-matching the *in silico *analysis against updated databases and accumulating data from the literature. The revised annotation contributed mostly to antigens which were consequently assigned as Intermediary metabolism and respiration (~75% of the re-annotated antigens). For example, of the 69 antigens which were re-annotated in this study, proteins Rv1461 and Rv1462, originally marked as hypothetical proteins, were found (both by domain analysis and literature search) to be part of the SUF machinery (mycobacterial system of [Fe-S] cluster assembly) identified by Huet and coworkers [145,146]. Likewise, most Usp proteins selected in this study were annotated as hypothetical proteins, and the functional assignment permitted to associate them with the stress protein family (see below). The potential contribution of ORFs with unassigned function to immunogenicity and vaccine is clearly demonstrated by the fact that of the 27 proteins in this category, 22% (6 antigens) are included in the list of 45 top-hits antigens (Table 3). All the six antigens are DosR regulon antigens and are also among the top-best T-cell antigens determined in this study (see below).

#### (2) Lipid metabolism

Accumulating information suggests that fatty acids, rather than carbohydrates, constitute the dominant carbon substrate utilized by Mtb during infection [147]. Bacteria isolated from lungs of infected mice were shown to oxidize preferentially fatty acids [148]. Moreover, the genes encoding fatty acid catabolic β-oxidation cycle enzymes are extensively duplicated in the genome [26,51]. In view of the nature of the dataset and the sources of information mostly related to aspects of the intracellular lifestyle of the bacilli and its survival, it is not surprising that the fraction of antigens related to lipid metabolism is as high as 16% (30 out of the 189 selected), compared to 6% (~240 antigens out of 3989) in the whole genome (p-value = 1.02 × 10^-4^) (Figure 2). Among the proteins involved in these processes, are many proposed vaccine candidates and drug targets. Several antigens have also shown promising cellular and humoral immune responses. Four out of the 30 proteins selected are among the 45 top-hit antigens. Basically, lipid metabolism can be divided into two main divisions:

##### (I) Lipid biosynthesis, modification and degradation

In this division are included 22 out of the 30 proteins in the category. Of the two discrete types of enzymes involved in fatty acid biosynthesis in bacteria, the type I and type II fatty acid synthases (FAS-I and FAS-II, respectively), condensing enzymes that catalyze the formation of carbon-carbon bonds, have received considerable interest, two of which are included in the 189 selected genes: *kasA *and *kasB *(Rv2245 and Rv2246, respectively) [26,149]. The *kasA-kasB *operon (β-ketoacyl-ACP-synthases) is necessary for synthesis of fatty acids, mycolic acid and polyketides, by initiating the subsequent rounds of acyl extension [150,151]. Few antigens selected in this study are related to biosynthesis of polyketides: a pks-associated protein (papA3, Rv1182) and a putative regulator of *pks *expression (annotated originally as a hypothetical protein, Rv1186c), the mycobactin biosynthesis operon (Rv2377c–Rv2386c, discussed below, in the section related to iron acquisition) as well as the polyketide non-peptide synthase (Rv0101c). Disruption of Rv0101 interrupts the virulence-related, cell wall constituent picothocerol dimycocereosate (DIM) biosynthesis, producing an avirulent strain with vaccine properties comparable to BCG [152]. The protein fadD26 (Rv2930) included in our selection, is a necessary part of the operon for biosynthesis of DIM [153], and its mutant is attenuated in mice and induces better protection than BCG when administered sc. [154]. The mutant is also associated with early attenuation of growth in macrophages [134]. Proteins involved in lipid modification include the Acyl-acyl carrier protein (ACP) desaturases, which convert saturated to unsaturated fatty acids. Three desaturases, desA1-A3, part of a multi-protein iron-enzyme complex (Rv0824c, Rv1094 and Rv3229c) were selected in this study. As for lipid degradation: fatty acid degradation genes are extensively duplicated in the genome (e.g. 36 paralogs capable of performing the first step, [26]), of which 6 were selected in this study (Rv0243, Rv3546, Rv2590, Rv3515c, Rv3139, Rv3140). Five proteins that are functionally related to lipid degradation are listed under either Intermediary metabolism and respiration (Rv1130 (*prpD*), Rv1131 (*gltA1*, citrate synthase), Rv0467 (*icl*) and Rv0211 (*pckA)) *or in PE and PPE proteins (Rv1169c). Rv1130, annotated originally as conserved hypothetical protein, is a homolog of *S. typhimurium prpD *and *mmge *– genes involved in β-oxidation of fatty acids, upregulated in macrophages [62], and essential for *ex vivo *intracellular growth. Rv0467 a member of the glyoxalate pathway, was one of the first genes reported to contribute to persistence [155-157]. Rv0211 encodes a gluconeogenetic rate-limiting enzyme – suggesting that fatty acids are in part converted into sugars via gluconeogenesis. In addition, Rv1169c (PE11), classified as a PPE and PPE protein, is one of numerous lipases which may be involved in host cell wall degradation, shows homology to triacylglycerol lipase, a paralog of which has been recently shown to be immunogenic [158,159].

##### (II) Lipid cell wall composition

Mycolic acids are high molecular weight α-alkyl, β-hydroxy fatty acids crucial for the architecture and permeability of the mycobacterial cell envelope. Free-standing lipids, lipoglycans, and proteins also intercalate within this complex. This layer is covered by a capsule consisting of polysaccharides, proteins and lipids. The consequences of these structural unique features are an extremely sturdy and impermeable cell envelope [151,160-162]. Mycolic acids containing glycolipids, in particular the prominent trehalose-6,6'-dimycolate (TDM), stimulate cellular and humoral immune response and granuloma formation [163]. Several gene products involved in mycolic acid biosynthesis and modification were selected in this study. Mycolyl transferases (Ag85 complex) are a family of proteins responsible for synthesis of cell-wall components – such as cord factor biosynthesis in Mtb (a dominant immunogenic and immunomodulatory structure necessary for maintenance of cell-wall integrity/virulence and an inhibitor of phagosome-lysozome fusion, [163,164]). Mycolyl transferases are also termed: fibronectin binding proteins, and have been shown to be involved in host entry. Two representatives are included in our selection, Ag85A (Rv3804) and Ag85B (Rv1886), both classical, well documented B-cell and T-cell immunogens and vaccine candidates. Another protein selected herein is *cmA2 *(Rv0503c), which plays a role in the modulation of the host immune response, by modifying the cord factor, hence forming a lipid molecule which suppress inflammation [165,166]. Finally, antigen Rv3130c, originally annotated as a hypothetical protein is actually *tgs *– member of a novel class of diacylglycerol acetyltransferases responsible for accumulation of triacylglycerol in Mtb as it enters a dormancy-like state in culture [167]. This antigen is constitutively over expressed in the epidemic Mtb of the W-Beijing lineage strain in comparison to non W-Beijing strains [168].

#### (3). Virulence, detoxification, adaptation

Inspection of Figure 2 reveals that antigens included in this functional category are enriched in the list of 189 antigens: 12% (23 antigens) as compared to only 2% (~80 antigens out of 3989) in the whole genome (p-value = 1.2 × 10^-5^). This is a consequence of the emphasis given to selection of antigens related to the pathogenesis traits. Of the 23 antigens, 6 are included in the list of 45 top-hit antigens. A total of 11 candidates in this category are stress proteins, belonging to the Universal stress protein (Usp) and the Heat shock protein (Hsp) superfamilies. The Usps encompass a conserved group of proteins involved in stress resistance, adaptation to energy deficiency, cell motility and adhesion [169]. The paradigm *usp *gene, *uspA *of *Escherichia coli *is transcriptionally activated by a large variety of stresses and is one of the most abundant proteins in growth arrested cells [170]. In our analysis, 6 out of the eight *usp *genes in the Mtb genome [171] were selected as vaccine candidates: Rv1636 (single Usp domain), Rv2028c, Rv3134c (a Usp domain followed by a typical C-terminal domain) and Rv1996, Rv2005c, and Rv2623 (harboring two Usp domains, and possessing a conserved ATP-binding motif and no signal sequence). Rv2623, Rv2005c, Rv2028c, and Rv3134c have been shown to be up-regulated under hypoxia [67], suggesting that they play a role in adaptation and survival of mycobacteria during growth arrest caused by oxygen limitation. The Hsp subfamily includes the extensively studied *M. tuberculosis *α-crystallin (*acr*) family-related proteins. The Mtb genome harbors 2 paralogs belonging to the *acr *family (hsp20/*acr*, Rv2031c and *acr2*, Rv0251). Rv2031c is regulated by the two-component regulatory system (DosR regulon), as detailed above, and is a latency stage disease marker [111]. While *acr *is induced by hypoxia or nitric oxide, *acr2 *is reported to be the protein most up-regulated under heat-shock as well as expressed under nitric oxide or during uptake by macrophages. Heat shock proteins assist in Mtb survival but also provide a signal to the immune response. *acr2 *has been shown to elicit a strong cellular response in cattle during early primary infection and thus has been suggested as a candidate for stage specific TB vaccine [172]. This protein is also tightly regulated by MprAB [173]. While analyzing immunogenicity of eight dormancy-regulon encoded proteins as DNA vaccines in a mouse model, Roupie and coworkers [25] have shown that Rv2031c induces the strongest Th1 response, and that mice persistently infected with Mtb developed an immune response against this gene. Both *acr *and *acr2 *have been documented as contributing to persistence [174]. Other Hsp proteins selected by this study are the endopeptidase Rv0384c (ClpB), Rv0350 – dnaK-Hsp70, and Rv0351-grpE (Hsp-70 cofactor), which are up-regulated in anaerobic stationary phase in *M. smegmatis*. PBMCs from TB patients and vaccinated individuals have been recently checked for cytokine profiles in response to DnaK [98]. To note, 4 out of the 6 candidates in the Virulence, detoxification and adaptation category included in the list of 45 top-ranking, are stress proteins (Rv1996, Rv2005c, Rv2031c and Rv2623).

Antigens Rv2006 and Rv3372 are part of the OstAB pathway, which was found to be essential for Mtb growth and virulence in mice [175,176]. This pathway involves the generation of Trehalose, a major cell-wall constituent of glycolipids. Rv3372 was recently reported to be an immunodominant antigen, inducing both humoral and cellular immune response. The enzyme was also recognized by patient's sera and BCG vaccinated donors; however no protection data was mentioned [177]. Rv1908c (KatG, a catalase/peroxidase/peroxinitritase) has been mostly studied in the context of resistance to isoniazid as well as in development and detection of MDR strains [178,179]. Intra-macrophage expression of *katG *is associated with growth and persistence of Mtb in mice and guinea pigs [180]; immunization with a multivalent combination DNA vaccine (containing the ESAT-6, MPT-64, MPT-63, and KatG) generated antigen-specific cell-mediated and humoral responses and elicited a strong protective response relative to BCG [122].

Another virulence-related family of proteins is Nlpc_p60, which belongs to the Firmicutes CHAP-related superfamily, consisting mainly of peptidoglycan hydrolases involved in cell separation. Recent evidences attribute a γ-glutamate-meso-diaminopimelate muropeptidase activity to Nlpc_p60 proteins [181]. Two members of this family, located in a single operon, are present in the Mtb genome, both selected herein: Rv1477 and Rv1478. It has been shown the *M. marinum *orthologs (*iipA *and *iipB*) are essential for virulence *in-vivo*, invasion and intra-cellular persistence in macrophages [182]. Moreover, it has been specified that the N-terminal sequence of Rv1477 is required for full virulence *in vivo *and in macrophages. In a recent study, Rv1477 was identified as an RpfB interacting protein (resuscitation promoting factor B, an important cell-wall hydrolase, see below), suggesting a role in cell division during reactivation/resuscitation [183].

Unlike the three functional categories described above, for which a major difference exists between their relative distribution in the list of 189 selected antigens as compared to that in the whole genome, a similar fractional distribution is observed in both lists with respect to the functional categories: Intermediary metabolism and respiration and the Cell wall and cell processes. The former includes 46 antigens, and obviously a thorough discussion of each of these antigens is beyond the scope of this paper, yet we would like to address three major clusters:

##### Iron acquisition

Iron acquisition-related gene products identified as candidates in this study, include a total of 16 antigens, 7 of which are classified as Intermediary metabolism and respiration proteins and are part of the SUF (mobilization of sulfur) machinery or iron storage proteins, while the remainder are siderophores-related proteins, classified either under the categories of Lipid metabolism or in the Cell wall and cell processes (ABC transporters, see below): (1) Siderophores: Mtb synthesize siderophores of the salicylate group, named mycobactins [90,184,185]. These are produced exclusively under iron limitation both as a membrane bound as well as soluble forms. The mbt-1 locus contains the siderophore core biosynthetic apparatus [90,91], members of which were selected by this study the non-ribosomal peptide synthetases MbtB and MbtE (Rv2383c, Rv2380c) two polyketide synthetases MbtD and MbtC (Rv2381c and Rv2382c), an isochorismate synthase (MbtI, Rv2386c), a hydroxylase (MbtG, Rv2378c) and the conserved protein MbtH (Rv2377c). MbtB mutants impaired in siderophore biosynthesis are unable to replicate in infected macrophages [186]. The *mbt*-1 locus genes were included in the Lipid metabolism category, given that these are polyketide biosynthesis components. Also selected as part of the iron acquisition apparatus are the ABC transporters IrtA and IrtB (Rv1348 and Rv1349, respectively) which actively transport the iron-siderophore complex. Inactivation of these genes drastically affects the ability of Mtb to replicate. *irtAB *mutant is attenuated for survival in infected macrophages and lungs of infected mice [187]; (2) The SUF (mobilization of sulfur) machinery: A locus of seven proteins was functionally identified by Huet and coworkers [145,146] as harboring the Mtb *suf *machinery – an exclusive mycobacterial system of [Fe-S] cluster assembly, probably essential for survival via its implication in bacterial resistance to low iron limitation and oxidative stress. The system is required for maturation of physiologically important metalloproteins. Sassetti and coworkers [61] defined this operon as necessary for *in-vitro *mycobacterial growth. Of this locus, our selection disclosed 5 components (Rv1461 (SufB), Rv1462 (SufD), Rv1464 (SufS), Rv1465 (NifU) and Rv1466. Rv1466 presents no homology with any documented *suf *gene but probably acts as a predicted metal-sulfur biosynthesis enzyme. Consistently, Rv1461 to Rv1466 are over-expressed in Mtb grown on low iron [94]. Moreover Rv1460, Rv1463 and Rv1464 have been described as stress response genes transcriptionally up-regulated in anaerobic stationary phase in *M. smegmatis*; (3) Iron storage proteins – bacterioferritin: Ferritins constitute the major non-heme iron storage proteins in animals, plants and microorganisms, playing an important role in ferric iron homeostasis. The Mtb genome harbors 2 paralogs: *bfrA *(Rv1876) and *bfrB *(Rv3841) both selected in this study. The *bfrB *antigen has been shown to be abundant under hypoxic conditions [188], a dominant T-cell antigen in infected mice [189] and antigenic in human disease [190].

Of the 16 candidate genes related to iron acquisition, only the two ABC transporters classified as Cell wall and cell processes category, Rv1348 and Rv1349, received relatively high score (qualitative scores of 7), yet the former was not included in the list of 45 top-hit candidates, because of a high number of transmembrane segments.

##### Nitroreductases

One of the activities induced in Mtb by hypoxia is the dissimilatory reduction of nitrate to nitrite which serves as an energy source as bacteria adapt to anaerobiosis [66,191]. Five nitroreductase-related proteins are included in the list of the selected 189 antigens, 4 of which are classified under Intermediary metabolism and respiration. The Mtb genome contains 2 loci homologous to prokaryotic respiratory NAR (nitrate reductase) genes [26]. The first-*narGHJI *is not represented in the list of 189 genes, while the second locus contains two antigens, both selected in this study: nitrate reductase narX (Rv1736), exhibiting strong similarity to the *narGHJI *cluster (yet probably inactive as a classical reductase [192]) and *narK2 *– a nitrite extrusion protein (Rv1737c), a member of the devR regulon (acting as a redox sensor [191] and an hypoxia induced antigen [67]). Three other selected ORF products, listed as putative nitroreductases, are part of a family of 8 unusual nitroreductases identified by Purkayashta and coworkers [79]: The hypoxia-induced *acg *(Rv2032, necessary for survival) and two paralogs (Rv3127 and Rv3131c), all harboring parts of the 5 nitroreductase order-conserved motifs identified [79]. Interestingly, of the five nitroreductase-related antigens selected, three (Rv2032, Rv3127 and Rv3131c) are included in the list of 45 top-hits.

##### Proteases

Although many proteases are documented as virulence factors in other pathogenic bacteria, little information is available regarding their role in *mycobacteria *pathogenesis. The Mtb genome harbors ~60 putative proteases [48], of which only 7 are included in the 189 list (all except one are classified under Intermediary metabolism and respiration). Among these are proteases being evaluated in vaccine studies: the classical Rv0125, a DegQ-like serine protease (HtrA-family), a construct of which with Rv1196c demonstrated protective efficacy equal to BCG in diverse animal models [135,193] and; the zinc-metalloprotease Rv0198c (pepO, an M13 peptidase resembling eukaryotic neprilysin [194]), which was evaluated as a vaccine candidate by Vipond and coworkers [96,97]. Rv2625c, originally annotated as a conserved transmembrane Ala and Leu-rich protein, is probably a zinc protease and part of the DosR regulon. Rv2869c is a homolog of the eukaryotic site two protease (*S2P*) controlling membrane compositions. In Mtb it has been recently found that the enzyme controls cell envelope composition, *in-vivo *growth and persistence [195]. Knockout mutants of Rv2869c exhibited reduced expression of genes related to lipid biosynthetic and degrading genes, including the resuscitation factor *rpfC*. Rv2224c was identified as a putative virulence gene by high throughput techniques and attenuation studies [61,76], and shown to be preferentially expressed in human macrophages and up-regulated after the onset of starvation. Similar to Rv2869c, Rv2224c may modify envelope composition or, alternatively, hydrolyze fatty acyl esters with mycobactericidal activity [196]. Another selected protease gene is the HslV protease Rv2110c (*prcB*, proteosome β-subunit), a core component of the proteosome, known to play a critical role in bacterial stress defense. None of the proteases selected in this study are represented in the list of 45 top-hits.

It is well established, that the characteristic mycobacterial cell envelope is a dominant feature of the biology of *M. tuberculosis *and other mycobacterial pathogens (as described above under lipid synthesis). Indeed, the second largest category which has a similar representation in the list of 189 selected antigens as in the whole genome, is the Cell wall and cell processes (a total of 38 antigens selected in this study). Most strikingly, 37% of the antigens under this functional category are included in the list of 45 top-hits candidates. This group contains three clusters of key antigens: the ESX secretion system, the resuscitation antigens and lipoproteins

##### The Esat-6 system (ESX)

The ESX is one of the 4 secretion systems which have been identified so far in *Mycobacterium tuberculosis*, and constitutes a major determinant in mycobacterial pathogenesis and immunogenicity. ESX-derived antigens have been described as immunogens in infected cattle [124,197], dominant early-phase T-cell antigens [102,106,119,127,136,142,198], putative subunit vaccine candidates [85,107,120], as well as potentiators of BCG vaccines [20]. These antigens have been also extensively studied as promising immunodiagnostic markers [84,85], and in mechanistic/regulatory studies ([199-201] and references therein).

The complete genome of Mtb H37Rv harbors five ESX copies (not necessarily identical, [202]). The ESX1 locus, the most well studied, is located in the extended region of difference 1 (extRD1) of the genome, and therefore absent from all vaccine strains of *M. bovis *BCG. The products of this system locus are suggested to modulate early events during infection (e.g. [102,199-201] and references therein) and involved in phenotypes as growth in macrophages, suppression of the macrophage inflammatory and immune response, phagosome maturation arrest [203] and virulence factor delivery. Sixteen ESX proteins are represented in the 189 selected antigens (9 of these are listed under the Cell wall and cell processes functional category). These include the classical well documented antigens: ESAT-6 (esxA, Rv3875) and 2 of its paralogs (the well studied vaccine candidate Rv0288 (esxH) and Rv1793 (esxN)), and CFP10 (esxB, Rv3874) and its paralog (esxG, Rv0287). In addition, the following ATPases were shown to be core components of the ESX system: Rv3871, Rv0282, Rv0283, and Rv0284, Rv3876 as well as the Ala-rich protein Rv3878 and Rv3879c, the transporters Rv0289 and Rv0290 and Rv0292, a transmembrane protein. Rv3616c (EspB) a recently identfied effector of this secretion system, was selected herein as well. In total, three ESX components (Rv0288, Rv1793 and Rv3875) are part of the 45 top-hit antigens.

##### Resuscitation promoting factors (Rpfs)

The Rpfs, secreted or membrane-anchored peptidoglycan/glycosyl hydrolases [204,205] were originally identified in *Micrococcus luteus*, promoting the recovery of bacteria from latency to a replicating phase [206]. In a later study the presence of similar autocrine growth factors in Mtb was reported [123,207]. Five Mtb gene products encoding Rpf homologs were identified (Rv0867c-*rpfA*, Rv1009-*rpfB*, Rv1884c-*rpfC*, Rv2389c-*rpfD *and Rv2450c-*rpfE*, [55]) and documented to have resuscitation activity [70]. Two strains of Mtb in which three *rpf*s were deleted were both attenuated in mice and did not reactivate in an *in vitro *reactivation assay [208]. Also, s strain lacking *rpfB *exhibited a delayed reactivation in a mouse dormancy model and reduced mouse lung colonization [71]. The Rpf proteins are secreted or membrane anchored, which renders them candidates for recognition by the host immune system. Affinity purified antibodies were shown to inhibit bacterial growth *in vitro *and it has been suggested that sequestration of Rpfs might also provide a means to limit/prevent bacterial multiplication *in vivo *[123,207]. When administered as subunit vaccines to mice, 4 out of 5 Mtb Rpfs were found to be highly immunogenic, eliciting both IgG1 and IgG2a responses (highest for Rv2389), T-cell proliferation and cytokine production. Vaccination of mice with RpfE (Rv2450c) results in significant protection against subsequent high-dose challenge with a virulent strain (in survival times and bacterial multiplication in lungs and spleen) to levels comparable to Ag85B and Esat-6 [88]. As mentioned, all the five known resuscitation promoting factors are included in the 45 top-ranking hits; three of which (RpfE, RpfB and RpfA) belong to the highest scoring group (GroupI, Table 3).

##### Lipoproteins

Lipoproteins are reported to affect both innate and adaptive immunity as well as bacterial *in vivo *growth and virulence. Of 48 predicted lipoproteins in the Mtb genome [209], 6 were selected in this study, and at least four are directly relevant for vaccine design. The classical 19 kDA antigen (Rv3763), is a well studied glycosylated lipoprotein acting as a phagocytosis stimulating adhesion [210] factor through TLR2 signaling in the macrophage, thus facilitating the bacilli persistence [209]. The lipoprotein LprG (Rv1411c) required for growth in mice [211], is a B and T-cell antigen [212], inducing protective T-cell response within the lungs of infected mice sufficient for early control of Mtb [140]. It is probably acting as a TLR-2 ligand, inhibiting MHC class II antigen processing [213,214]. In a more recent study, a fusion protein of Rv1411c with ESAT-6 (where Rv1411c acts as a natural adjuvant) was constructed, resulting in protection of mice exposed to low dose aerosol challenge [214]. RpfB (Rv1009), the only lipoprotein in the resuscitation factor family, is another prominent representative described above. In addition to these clusters of antigens, a promising cell wall antigen is the secreted T-cell antigen TB8.4 (Rv1174c). This protein, detected in human macrophages and sputum of TB patients [215], affects growth of *M. bovis *BCG in human macrophages and mice [216], probably via its role in both resistance to intracellular stress and adaptation to hypoxia [217]. The Rv1174c knockout mutants are attenuated *in vivo *and Rv1174c-derived peptides were found to resuscitate one year old cultures [218]. Moreover, immunization with either plasmid DNA or the Rv1174c recombinant protein induces a strong and protective T-cell response in mice [105].

Another functional cluster of proteins is the unique surface-associated PE & PPE family, which encompasses ~4.5% of the Mtb genome [219-222] and a similar percentage in the 189 selected list (Figure. 2). The PE family harbors a Pro-Glu motive at their N-terminus (positions 8 and 9 in a 110 residue long conserved domain). A subgroup of the PE family is the PE-PGRS, where the PE domain is followed by multiple tandem repeats of GGA or GGN [223,224]. In the PPE family (ProProGlu N-terminal motif, positions 7–9 in a 180 residue conserved domain) the repeat domain is followed by a variable C-terminus [221]. Originally members of this family were used as strain differentiation markers [225]. PE/PPE genes are widely present in pathogenic bacteria yet missing in non-pathogenic species, and therefore are assumed to fulfill important functions related to survival within different environmental niches. Indeed, the first evidence came from the observation that inactivation orthologs of the PE gene product *wag22 *in *M. marinum *results in a replication defect in macrophages and decreased survival in granulomas [226]. Certain PE/PPE proteins play a role in immune evasion and antigenic variation [26,219,223,227], others are virulence related [224,226,228,229]. Evidence that at least some PE_PGRS genes are antigens expressed during infection in the host comes from serological studies. Many of the PE/PPE proteins elicit strong immune response [104,159,227,230-232]. Of the antigens selected in this study, 10 belongs to the PE/PPE family (equivalent to its fraction in the genome), of which three (Rv1169, Rv3347c and Rv3873) are included in the top-ranking 45 antigens (see Additional file 1: List of 189 selected antigens, and Table 3), most of them having particular indications as for their involvement in virulence and/or immune response, as follows: PPE55 (Rv3347c) is recognized by antibodies elicited during sub-clinical infections of guinea-pigs and could be applied to the differentiation between latent and incipient TB [233]. PPE protective subunit (Rv3812) was recently identified as T-cell antigens with vaccine potential [103]. Rv1169c elicits a strong but differential B-cell response among different categories of TB patients [234]. PPE68 (Rv3873) which is a member of the RD1 region, was shown to act as both a T-cell and B cell antigen in mice [106] and is also a constituent of the ESX secretion system, as are the selected PE5 (Rv0285) and PPE4 (Rv0286).

### Distribution of immunopotent T-cell antigens

Inspection of the combined weighed scores of experimental data and predictions in the T-cell immunogenicity category revealed that 29 antigens harbor the highest scores in this category (a total quantitative score 3 or 4). Of these, 70% (20 antigens) are included in the list of 45 top-ranking candidates, where 4 of which are proteins of unknown function. More strikingly, all 6 hypothetical proteins in the list of the 45 top-ranking antigens are immunopotent T-cell candidates (Rv0079, Rv1813c, Rv2030c and Rv2627c are part of the 29 top best T-cell antigens, while Rv1738 and Rv2628 are documented in literature sources on individual antigens). The distribution of the remaining immunopotent T-cell antigens is comparable, ~2–3 antigens per functional category. In addition, among the 29 immunopotent antigens are also included 3 out of the 8 antigens classified as Intermediary metabolism and respiration in the list of 45 top hits, as well as 2 of 4 Lipid metabolism categorized proteins, the well documented classical Ag85A (Rv3804c) and Ag85B (Rv1886c).

The 20 most immunopotent T-cell antigens included in the list of 45 top-hit candidates, can also be examined with respect to their distribution among different classes/phases (as assigned for each ORF product, see Additional file 1: List of 189 selected antigens). Out of the 20 antigens, 11 are DosR regulon members (Rv0079, Rv1196, Rv1813c, Rv2029c, Rv2030, Rv2031c, Rv2032, Rv2627c, Rv3127, Rv3130c, Rv3132); 4 are classical vaccine antigens (Rv1886c, Rv3804c, Rv1980c, Rv2875), one resuscitation antigen (Rv0867c), one putative reactivation antigen (Rv0288) and 3 are listed under "others" (Rv1908c – *katG*, Rv2780-*ald*, Rv3873-*PPE68*, an ESX1 component). Search for T-cell immune response data originating from literature documentation on individual antigens in the list of 189 selected genes identified at least 15 additional immunopotent candidates, 9 of which are included in the top list of 45 antigens (the resuscitation factors – Rv1009, Rv1884c, Rv2389, Rv2450; Rv1174c-a low Mw T-cell antigen and the DosR regulon antigens – Rv2006, Rv1738, Rv1733c, Rv2626c and Rv2628 studied by Leyten and coworkers [24] & Roupie and coworkers [25]). Interestingly, the most potent T-cell antigens, if estimated solely on the *in silico *predictions of CTL binders conducted herein are ~10 antigens harboring 80–200 epitopes recognized by all 12 supertypes (in decreasing order Rv0101-*nrp*, Rv2380c-*mbtI*, Rv0284-*ftsk*, Rv2383c-*mbtB*, Rv2006, Rv2590-*fadD9*, Rv3347c-*PPE55*, Rv0450c-*mmpL4*, Rv1997-*ctpF*, and Rv1736c). It may be of interest to evaluate experimentally these promising antigens for their activity as T-cell antigens.

## Conclusion

This study illustrates the process of screening the complete Mtb genome, aiming at identifying and selecting potential vaccine candidates. The screen was implemented to discern genes covering all phases of the infection, which would, in turn, contribute either to construction of a multi-stage vaccine or to the design of a subunit vaccine supplemental to the BCG vaccine, solely directed to the early-phase events of the infection. The strategy designed for rational candidate selection was applied onto a knowledge dataset which compiled numerous sources of relevant published experimental information. The literature-based dataset includes ~1200 antigens, for which evidences on aspects relevant to vaccine and/or virulence traits of the bacteria, existed (classified into 11 categories). An initial reduction to a list of 344 antigens was conducted, based on: (1) a preliminary total score calculated from the number of positive signs in the various categories, resulting in 143 antigens having a relatively high score of 3–8; (2) a subsequent selection from the remaining 201 antigens (scoring 1–2), by manual curation of specific sources of information related either to vaccine or pathogenesis; resulted in additional 46 antigens (interestingly, only 4 of the 46: the classical Rv1908c, Rv1926c, Rv1980c, and the ESAT protein Rv1793 were eventually included among the final list of 45 top-hits antigens (Table 3)). These filtering steps generated the list of 189 selected vaccine candidates, on which further extensive bioinformatic and immunoinformatic analyses were conducted. Of the 189 antigens in the list, only 34 were previously described in the literature as putative vaccine candidates. These 34 known antigens represent over 80% of the total number of such antigens listed in the previous step of the selection process (see Figure. 1). Among these 34 antigens, at least 5 are under clinical trials (the classical antigens: Ag85A (Rv3804c) and Ag85B (Rv1886c), ESAT-6 (Rv3875); mtb72f: Rv0125 and Rv1196), 10 were used in protective studies in animal models and 19 are candidates eliciting a strong immune response.

Examination of the distribution of the 189 selected antigens among whole genome-defined functional categories (Additional file 1: List of 189 selected antigens) discloses an enrichment of proteins related to lipid metabolism as well as virulence and stress proteins, in comparison to their fraction in the genome. Also, the *in silico *analyses contributed to the updating of the annotation, resulting in a dramatic decrease in the number of proteins of unknown function in comparison to their fraction in the whole genome. The comprehensive dataset was then employed to rank the selected antigens, by applying a ranking scheme based on the assignment of both qualitative and quantitative scores to each of the antigens, in the 14 criteria employed. This ranking enabled to down select a list of the best 45 candidates, denoted as top-hit antigens (Table 3).

Inspection of the proposed list of 189 antigens provided in Additional file 1: List of 189 selected antigens reveals representatives of all classes/phases of the infection, including early phase well-documented classical antigens as well as 14 other early phase antigens. It is worth noting that all five known resuscitation antigens (RpfA-E) are included and furthermore, all 5 are among the 45 top-hit antigens (Table 3). The DosR regulon comprises of ~50 genes [52,53,235] (1.3% of the genome), 36 of which are represented in the list of 189 antigens, and 20 in the list of 45 top-ranking antigens (Additional file 1: List of 189 selected antigens and Table 3). Overall, an enrichment of DosR-regulated and reactivation/resuscitation antigens is observed in the list of 45 top-ranking antigens (74% as compared to 36% in the list of 189 selected antigens). This enrichment is obviously in part a consequence of the criteria used for selection, which emphasize late stage phases of the bacteria life cycle. In light of this observation, the result that early stage classical antigens (such as Rv1886 (Ag85B), Rv3804 (Ag85A) and Rv0288 (esxH)) are found among the highest scoring candidates (GroupI and GroupII of the top-ranking 45 antigens, Table 3), provides some validity to the methodology used in this study to map antigens pertinent to all phases of the disease. Murphy and coworkers [18] recently suggested a list of 118 putative dormancy drug targets; interestingly, 27 of these overlap with our 189 selected candidates. Out of these 27 antigens, 16 are part of the DosR regulon, and 11 are included in the list of 45 top-hit antigens. This extent of overlap is a reflection of the facts that: (a) our datasets are not identical; (b) in our analysis, considerable weight was assigned to the immunogenic and virulence potential of each ORF in the genome.

The list of top-ranking 45 antigens (Table 3) could therefore provide a platform for choosing combinations of representatives from the late-stage antigens, which, together with the classical antigens, may contribute to an improved protection as compared to current vaccines based on the early-phase directed BCG. Inclusion of DosR antigens in a future vaccine may be essential in view of the apparent limited immune response induced by the BCG to the DosR regulon proteins, indicating that the vaccine strain probably does not express these late stage antigens [118] moreover the DosR regulon was shown to be constitutively expressed in the Beijing epidemic strain [168], further emphasizing the relevance of these late stage proteins.

Guided by the analysis described in this study, and based on the list of 45 top-ranking antigens (Table 3), we have recently generated a novel rBCG vaccine denoted AERAS-407, which allows over expression of the following selected antigens from this list: (1) the classical antigens Ag85A and Ag85B; (2) DosR regulon genes, the expression of which was induced via overexpression of *dosR*; (3) the resuscitation antigens Rv0867c, Rv1884c, and Rv2389c. In addition the AERAS-407 vaccine includes the reactivation antigen Rv3407 [95]. This novel rBCG vaccine candidate has been produced and is currently under experimental evaluation.

## Competing interests

The authors declare that they have no competing interests.

## Authors' contributions

AZ and NA performed most of the analysis and participated in writing the draft ms. JF participated in discussions during the study and was responsible for constructing the rBCG based on the analysis. JCS participated in discussions at all stages of the study and co-wrote the final ms. AS conceived the study, participated in the analysis and co-wrote the manuscript. All authors read and approved the final manuscript.

## Pre-publication history

The pre-publication history for this paper can be accessed here:



## Supplementary Material

Additional file 1 **List of 189 selected antigens**. (a) In bold and underlined: top-ranking antigens included in the list of 45 candidate genes. (b) The Gene name and annotation are based on the data deposited at the NCBI [GenBank: AL123456]. In square brackets: updated gene name, annotation, and functional category, resulting from the analyses conducted in this study. (c) The functional categories are as provided by Cole and his coworkers [26], and the assignment of a functional category to each ORF product is according to the Tuberculist database [47], unless an updated category is assigned (in square brackets), according to the re-annotation performed in this study (see (b)). (d) The classes/phases of Mtb infection assigned to each selected ORF (see "Methods"). Click here for file

Additional file 2 **Raw data of the 189 selected antigens**. The data (a '+' sign or an experimental value) extracted from global and particular analyses are provided, for each category and references therein (see Table 1). In addition, the qualitative and quantitative scores for each antigen in each of the categories are given. Click here for file

Additional file 3 **Qualitative scores of the 189 selected antigens**. The qualitative score for each of the 189 selected ORF in each of the categories is provided. A "+" sign is indicated if data exists at least in one study in that particular category (see Table 1 for data resources), and the arithmetic sum of all "+" for a particular ORF is given in the right column as the total qualitative score (Qual_Total). (a) The Gene name and annotation are based on the data deposited at the NCBI, [GenBank: AL123456]. In square brackets: updated gene name and/or annotation, resulting from the analyses conducted in this study. For the complete data values of the 189 selected antigens in each of the categories, see Additional file 2: "Raw data of the 189 selected antigens". Click here for file
